# A High Resolution Capacitive Sensing System for the Measurement of Water Content in Crude Oil

**DOI:** 10.3390/s140711351

**Published:** 2014-06-25

**Authors:** Muhammad Zubair Aslam, Tong Boon Tang

**Affiliations:** Department of Electrical and Electronic Engineering, Universiti Teknologi PETRONAS, Bandar Seri Iskandar, 31750, Tronoh, Perak, Malaysia; E-Mail: mzubair.ciit@yahoo.com

**Keywords:** capacitance sensor, interface circuit, water content, crude oil

## Abstract

This paper presents the design of a non-intrusive system to measure ultra-low water content in crude oil. The system is based on a capacitance to phase angle conversion method. Water content is measured with a capacitance sensor comprising two semi-cylindrical electrodes mounted on the outer side of a glass tube. The presence of water induces a capacitance change that in turn converts into a phase angle, with respect to a main oscillator. A differential sensing technique is adopted not only to ensure high immunity against temperature variation and background noise, but also to eliminate phase jitter and amplitude variation of the main oscillator that could destabilize the output. The complete capacitive sensing system was implemented in hardware and experiment results using crude oil samples demonstrated that a resolution of ±50 ppm of water content in crude oil was achieved by the proposed design.

## 1. Introduction

Determination of water content is an important issue in grading crude oil. Conventional industrial approaches include distillation, centrifugation and electrical dewatering [1]. These methods require expensive analytical tools and complex processes. Alternative methods have also been proposed based on density, microwave, ray, capacitance and shortwave absorption measurement principles, albeit they are limited to high water concentrations (≥10%) [2,3].

To grade a higher purity of crude oil, a sensing system with a higher resolution is required. Among all, a capacitive sensing system is a promising choice of sensor based on cost, stability, design flexibility, signal-to-noise ratio and potential of system level integration. In particular, the system benefits enormously from recent advances in silicon fabrication technology that allows accurate and repeatable fabrication of miniature capacitance sensors. The application of capacitive sensing system now includes pressure sensing [4], position sensing [5], humidity sensing [6], liquid level sensing [7], void fraction measurement in two-phase flow [8–13], and human blood cells measurement [14].

The concept of *void fraction measurement* is adopted in this work to quantify the water content. The concept is not limited to gas-liquid but also applicable to liquid-liquid. In the simplest sense, the volume of a liquid/gas is measured by estimating the dielectric permittivity of a pipeline with a capacitance sensor. In [8], oil fraction was measured in two phase flows (oil-water) by using a single pair of concave electrodes mounted outside of pipe walls. The system was tested at frequency of 2 MHz to minimize the shielding effect. The capacitance measurements were subsequently converted into DC voltage using a demodulation circuit. In [9], the electrodes were mounted both inside and outside of pipe walls. The system was tested with different two phase flows, *i.e.*, water-oil, oil-gas and water-gas. The system operating frequency was 1 MHz. Another type of sensor design used a ring and a concave electrode as a capacitance sensor [10]. It measured the flow rate from the time-varying capacitance readings. In [11], helical electrode capacitance sensor was proposed for water-air two phase measurements. *Phase shift* as a result of capacitance change was measured with an input signal of 1 MHz by an interface circuit. The two phase components were water and gas in stationary state [12].

There are various designs of interface circuit for capacitive sensing and they can be categorized, into the following type:

- Charge amplifier that converts measurements into DC voltage [15,16].
- Modified Schering Bridge network [17].
- Switch capacitor circuit [18].
- Direct interface to microcontroller without any analog circuit in signal path [19].
- Resonant circuit with capacitance sensor acting as the key component determining resonance frequency such that different capacitances will result different resonance frequencies [20].

All the aforementioned circuits provide measurements adequate for general crude oil grading. To achieve higher precision, a charge amplifier variant capable of capacitance to phase angle conversion should be used [21]. The circuit uses a compensating signal to permit a gradual phase shift in response to the change in capacitance, in place of the abrupt 180° turnover produced by the conventionally compensated charge amplifier. This enables the circuit to be sensitive even to sub-femto farad changes [21]. However it comes with some limitations such as any phase jitter and amplitude variation of the input signal (the main oscillator) can cause destabilization of its output.

In this paper, we will exploit this capacitance to phase conversion circuit for the application of ultra-low water content in crude oil measurement. In addition, we introduce a differential sensing technique to overcome the aforementioned limitations for more stable output readings. We also propose a simple phase readout mechanism to eliminate the need of an analogue-to-digital converter (ADC).

## 2. System Design

This section describes our capacitance sensor design and the corresponding interface circuit design.

### 2.1. Capacitance Sensor

**Figure 1.**
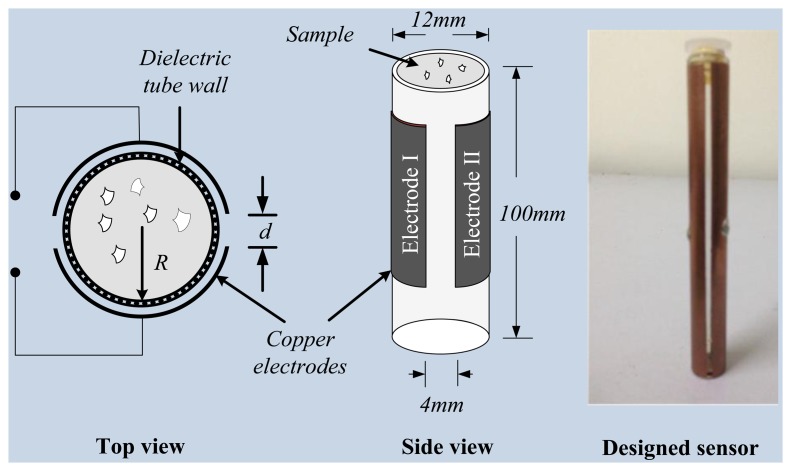
Design of the semi-cylindrical capacitance sensor.

Two capacitance sensors (one for measurement and one as reference) with identical specifications have been carefully designed, simulated with COMSOL Multiphysics and developed for this particular application. Each sensor has a pair of semi-cylindrical electrodes made from soft copper adhesive foil. The electrodes are mounted on the outer side of a glass tube, as shown in Figure 1. This allows non-intrusive measurement about crude oil sample under test. The diameter of tube is 12 mm and length is 100 mm. The length of each electrode is 90 mm and the minimum spacing between electrodes is 4 mm. The capacitance sensor has an initial capacitance of 10.3 pF with pure oil as dielectric material.

The capacitance of the sensor depends on the dielectric permittivity of medium between the pair of electrodes. The actual capacitance (*C*_a_) due to crude oil sample is in series with glass tube wall capacitance (*C*_w_). Therefore the total capacitance (*C*_t_) of the sensor can be expressed as:

(1) $$C_{\text{t}}=\frac{C_{\text{a}}C_{\text{w}}}{C_{\text{a}}+C_{\text{w}}}$$

**Figure 2.**
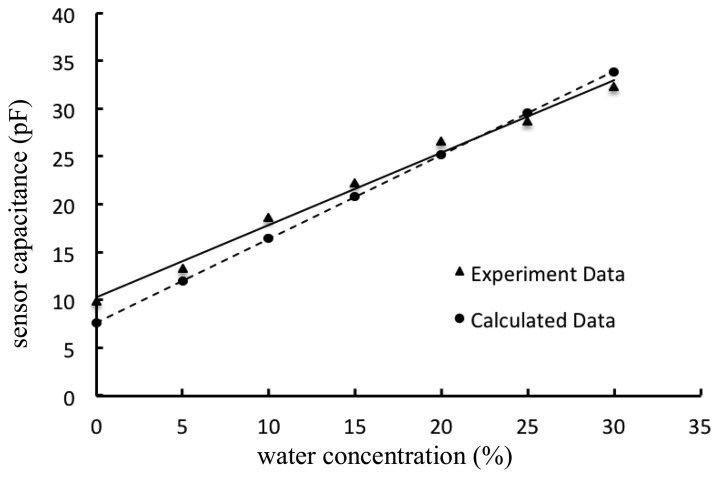
Sensor capacitance at different level of water concentration.

The capacitance of glass wall (*C*_w_) is dependent on the dielectric material of the glass and is a constant, while the actual capacitance (*C*_a_) is proportional to the dielectric constant of the crude oil sample filling the glass tube. The effective permittivity (ε_a_) of two liquids, which mainly depends on volume percentage of two phases in tube, is given by [9]:

(2) $${ɛ}_{\text{a}}=\frac{V_{\text{w}}{ɛ}_{\text{w}}+V_{\text{o}}{ɛ}_{\text{o}}}{V_{\text{t}}}$$

where *V*_w_ is the volume of water in glass tube, ε_w_ is the dielectric permittivity of water, *V*_o_ is the volume of crude oil, ε_o_ is the dielectric permittivity of oil, and *V*_t_ is the total volume of sample. The capacitance of the sensor having two semi-cylindrical electrodes of same size, separated by a distance can be written as [13]:

(3) $$C_{\text{a}}=\sum_{i=0}^{n}2{ɛ}_{\text{o}}{ɛ}_{\text{a}}A_{\text{e}}\times \left[\frac{1}{d+(i-1)\Delta d}\right]+\frac{{ɛ}_{\text{o}}{ɛ}_{\text{a}}A_{\text{e}}}{2R}$$

where *A*_e_ is the unit area of the electrode, *ε*_o_ is the dielectric permittivity of free space, ε_a_ is the dielectric permittivity of sample inside glass tube, *d* is the minimum distance between the electrodes, *R* is the radius of the tube and Δ*d* is an increment distance between semi-cylindrical concave electrodes. The calculated capacitance values with respect to water concentration can be obtained from Equations (2) and (3). Using an inductance (L), capacitance (C) and resistance (R) analyzer—a LCR meter (Agilent E4980A, Agilent Technologies, Santa Clara, CA, USA) as a reference, the capacitance of the sensor was measured at different levels of water concentration in crude oil. The experimental and calculated results are shown in Figure 2. The calculated sensor capacitance change is 0.876 pF for 1% of water concentration, while the actual measured capacitance change is 0.757 pF. Both graphs have high linearity (*R*^2^ ≥ 0.99), hence making it straightforward to estimate the actual water concentration (in percentage units) based on capacitance change measurements.

### 2.2. Interface Circuit

**Figure 3.**
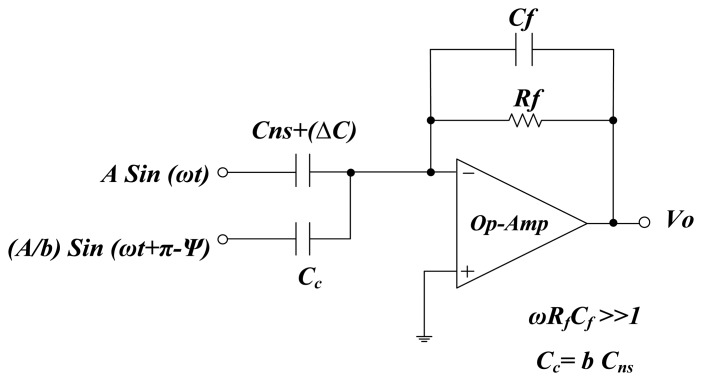
Capacitance to phase conversion circuit [21].

**Figure 4.**
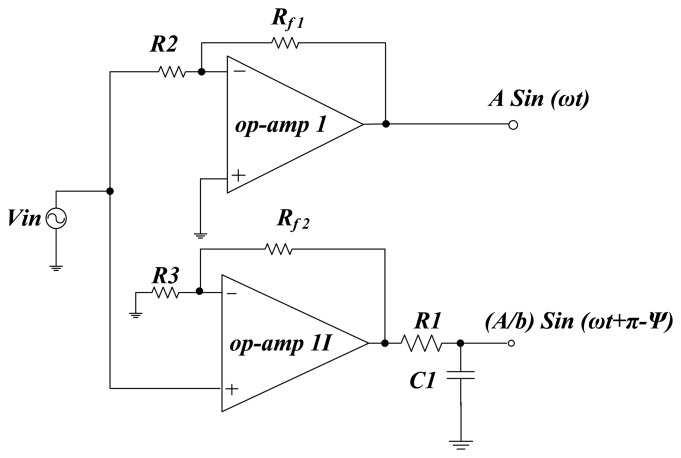
Circuit to generate compensating driving signals.

Figure 3 shows the interface circuit of the proposed system that is based on the configuration of a capacitance to phase angle conversion circuit [21]. The parameter *C*_ns_ is the nominal capacitance value of the measurement sensor, *C*_c_ is the compensation capacitance, *C*_f_ is the feedback capacitance and *R*_f_ is the feedback resistor. The two input driving signals, *i.e.*, *A* sin(ω*t*) and (*A*/*b*)sin(ω*t* + π − Ψ) are generated by a circuit shown in Figure 4. The phase shift of 180° is produced by op-amp I and the output signal of op-amp II is shifted by a RC low pass filter. The angle of phase-shifted signal depends on desired value of angle Ψ. The value of Ψ defines the sensitivity and operating region, and can be calculated by RC low pass filter formula, *i.e.*, Ψ = −arctan(ω*R*_1_*C*_1_) where *A* is the amplitude of the signal and (*A*/*b*)sin(ω*t* + π − Ψ) is the compensating signal.

The capacitance to phase angle conversion circuit shown in Figure 3 works like a summer amplifier. Therefore generalized equation for this circuit can be written as:

(4) $$\frac{V_{\text{o}}}{A}=-\frac{\left(C_{\text{ns}}+\Delta C\right)}{C_{\text{f}}}\sin\omega t-\frac{C_{\text{c}}}{bC_{\text{f}}}\sin(\omega t+\pi -\Psi )$$

where *C*_c_ = *bC*_ns_, then from Equation (4):

(5) $$\frac{V_{\text{o}}}{A}=-\frac{1}{C_{\text{f}}}[(C_{\text{ns}}+\Delta C)\sin\omega t+C_{\text{ns}}\text{sin}(\omega t+\pi -\Psi )]$$

Equation (5) shows the relation between input driving compensating signal and the output voltage of charge amplifier. After applying geometric properties and expansion of sin Ψ and cos Ψ in first two terms of power series, Equation (5) can be simplified as:

(6) $$\frac{V_{\text{o}}}{A}=-\frac{1}{C_{\text{f}}}\sqrt{{\left(\Delta C_{\text{ns}}+0.5\Psi^{2}C_{\text{ns}}\right)}^{2}+{\left({\Psi \text{C}}_{\text{ns}}\right)}^{2}}.\cos(\omega t-\theta )$$

and:

(7) $$\theta ={\text{tan}}^{-1}\left(\frac{0.5\Psi^{2}C_{\text{ns}}+\Delta C}{\Psi C_{\text{ns}}}\right)$$

**Figure 5.**
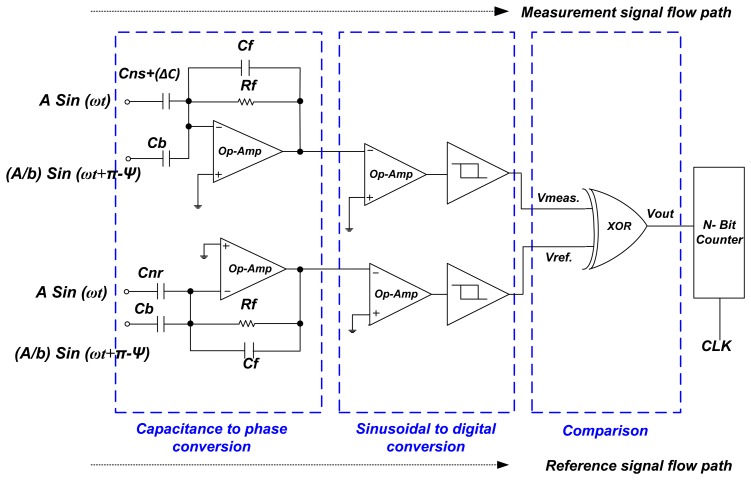
The complete interface circuit of the system.

The phase shift in output signal of charge amplifier can be calculated using Equation (7) and amplitude of the signal can be calculated with Equation (6). The proposed interface circuit has three main sections, as shown in Figure 5. The first section is the capacitance to phase conversion section. In this section, two capacitance to phase converters are used; one is used for measurement sensor (*C*_ns_) and other for reference sensor (*C*_nr_). The purpose of using dual capacitance to phase converters is to enable the removal of any phase jitter and amplitude variation of the input signal (the main oscillator) which may cause destabilization at the interface circuit output.

**Figure 6.**
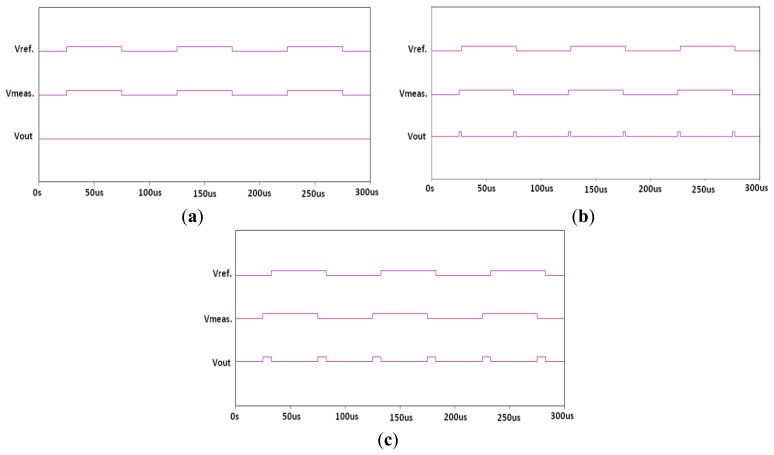
The PSPICE simulation results for Ψ = 0.35. The output of Exclusive-OR (XOR) gate when (**a**) water content is 0 ppm; (**b**) water content is 50 ppm; and (**c**) Water content is 200 ppm.

**Figure 7.**
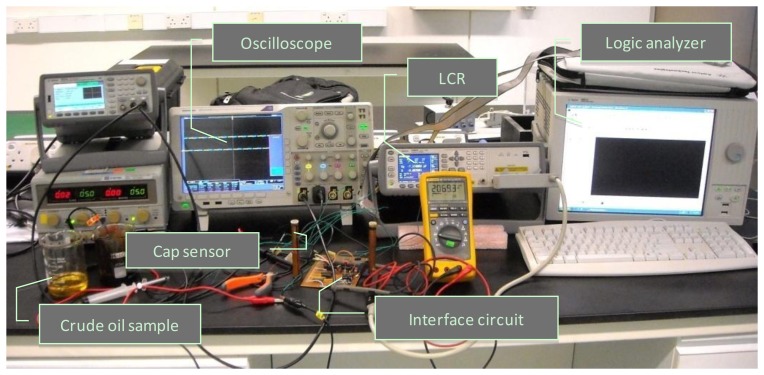
The setup used for experiment with actual crude oil samples.

The Exclusive-OR (XOR) gate outputs the phase difference between both sensor signals. Figure 6 illustrates example PSPICE simulation waveforms (Cadence Design Systems, Inc., San Jose, CA) for different water concentration levels. Figure 6a shows the XOR gate outputs when the water content is 0 ppm. At this point the output is always zero because the capacitance of both sensors is equal. When the water content is 50 ppm, the XOR gate outputs a narrow pulse stream, as shown in Figure 6b, and a wider pulse stream when the water content is increased to 200 ppm as in Figure 6c. An 8-bit counter is further used for measurement of the pulse width. The final outputs are analyzed using an Agilent 16801A logic analyzer (Agilent Technologies). The experimental setup is shown in Figure 7.

## 3. Results and Discussion

**Figure 8.**
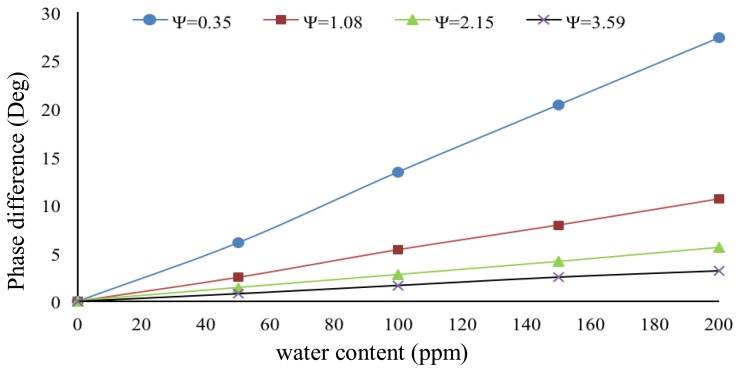
The simulation results for different values of Ψ.

The interface circuit is simulated using PSPICE to study the sensitivity of our sensing system at various values of Ψ. The simulation results are shown in Figure 8. The results show that lower values of Ψ give higher sensitivity but at reduced linearity. The selection of Ψ therefore depends on the range of water content level need to be quantified in the application.

A prototype of the proposed capacitive sensing system has been implemented on a Vero board to confirm the system design. Discrete components were used although future implementation of the interface circuit would be monolithic integrated circuit (IC) based. For current implementation, a UA3140 IC was selected as the op-amps and 74lS590 as the 8-bit counter. The XOR gate drove the counter. The clock frequency of the counter was 10 MHz and the input sinusoidal signal frequency was 10 kHz with amplitude of 1 V. The width of output pulse by the XOR gate was set to vary from 0% to 25% in response to phase shift in range between 0° to 90°.

In order to evaluate the system performance, both sensors were first filled with pure crude oil before water was gradually added through a syringe. The crude oil sample was obtained from the Dulang Oil Field, Malaysia and was processed using the industrial standard distillation procedure (ASTM D4006 [22]) to make it water free. To control the water content precisely, a micro syringe with a minimum volume size equivalent of 50 ppm water was used. This 50 ppm of water content translates to a 4.7 fF change in capacitance value of the sensor. Based on Figure 8, the circuit sensitivity is about 1.5°/fF (at Ψ = 0.35) and can be varied by selecting different values of Ψ.

**Figure 9.**
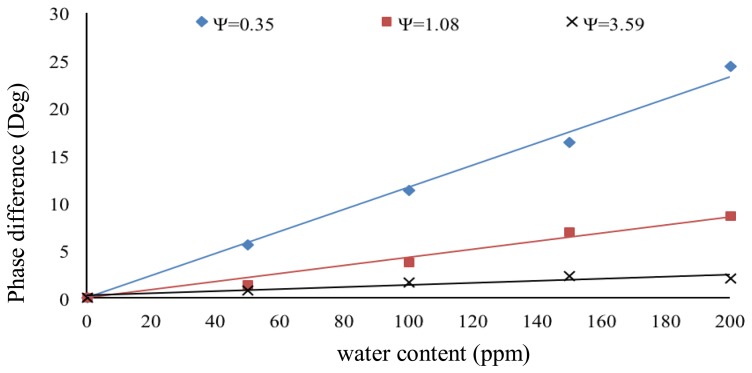
The experimentally measured results for different values of Ψ.

The experimental results are summarized in Figure 9. As mentioned before, the sensitivity of the interface circuit depends on the selected value of angle Ψ. A trade-off must be made between sensitivity, measurement range and linearity. For our particular application, we needed a high sensitivity to detect very small water content values in crude oil. Therefore an optimal value of Ψ (0.35 in present case) was selected to obtain the desired results. Some practical non-idealities and limitations may affect the overall performance of the system. These include sensor properties, interface circuit or other reasons. At 50 ppm of water content, the value of Δ*C* was 4.7 fF. Referring to Table 1, the circuit has a sensitivity of 8.60 ppm of water content per degree of phase change at Ψ = 0.35°. The interface circuit may detect less amount of water content (sub 50 ppm), albeit limited by the resolution of the micro syringe we used.

As mentioned above, the capacitance to phase conversion circuit has a sensitivity of 1.5°/fF. The phase shift can be from 0° to 90°, mapping to an equivalent output pulse width of 0% to 50%. Since the maximum phase angle is 90° and the sensitivity is 1.5°/fF, the equivalent maximum capacitance change is 90/1.5 fF = 60 fF. As each 50 ppm is equal to a 4.7 fF capacitance change, therefore the maximum detectable water content is 50 × 60/4.7 ppm = 638.3 ppm. Therefore, the range of water content our system can measure is between 50 ppm and 638 ppm. The overall system specifications and characteristics are summarized in Table 2.

## 4. Conclusions

A capacitive sensing system based on capacitance to phase angle conversion has been proposed to measure high purity crude oil. The system design has been validated using software simulation and hardware implementation. In testing with actual crude oil samples, the system could achieve a resolution of 50 ppm water content and higher resolution should be achievable. A differential sensing technique was introduced into the proposed system to address the issues of jitter noise and amplitude variation of the main oscillator. As compared with the conventional methods, the proposed system offers a simple and portable solution to grade high purity crude oil.

## Figures and Tables

**Table 1.** Summary of the proposed system performance based on Figure 9.

**Values of Ψ**	**Sensitivity as Ratio of Water Content and Phase Difference (ppm/°)**	**Correlation Coefficient (*R*^2^)**
0.35	8.60	0.99
1.08	23.47	0.98
3.59	78.74	0.84

**Table 2.** Summary of the system specifications and characteristics.

**Sensing Section**		**Interface Circuit**	
Sensor geometry	Single electrode paired semi-cylindrical sensor	Methodology of interface circuit	Capacitance to phase angle conversion
Material of electrodes	Copper	Testing	PSPICE simulation and experimental
Electrodes geometry	Length: 90 mm, Spacing: 4 mm, Thickness: 65 μm	Measurement resolution	Up to 1 fF
Diameter of the sensing tube (external)	12 mm	Circuit sensitivity	1.5°/fF (at Ψ = 0.35)
Length of glass tube	100 mm	Input excitation	10 kHz, 1 V_p_
Maximum capacity of the tube	10 mL	–	–
Standing capacitance	10.3 pF	–	–

**Overall System**			

Detection resolution		±50 ppm	
Detection range		Between 50 ppm and 638 ppm	
Fluid used		Dulang oil field sample, Malaysia	
Application		Petroleum industry	
